# Left atrial thrombus due to transseptal catheterization simulating solid mass of right atrium

**DOI:** 10.1186/s13019-017-0628-y

**Published:** 2017-09-01

**Authors:** Jae Hang Lee, Ji-Hyun Kim, Jin-Ho Choi, Eung-Joong Kim

**Affiliations:** 1 Department of thoracic and cardiovascular surgery, Dongguk University Ilsan Hospital, Siksa-dong, Ilsandong-gu, Goyang-si, Gyeonggi-do 410-773 South Korea; 2 Cardiovascular center, Dongguk University Ilsan Hospital, Goyang, Gyeonggi South Korea

**Keywords:** Atrial septum, Catheterization, Thrombus, Cardiac tumor

## Abstract

**Background:**

Transseptal catheterization has been popularized through ongoing advances in percutaneous procedures, but related complications are known to occur.

**Case presentation:**

A 72 year-old female was admitted with left-sided weakness. In the course of various exams, a rounded and smooth-surfaced solid mass of right atrium was identified. However, a septal aneurysm associated with left atrial mural thrombus was evident intraoperatively. Given that percutaneous transseptal mitral valvotomy had been done 7 years previously, a causal relationship is likely.

**Conclusions:**

Prior cardiac intervention should be considered in patients presenting with mass lesions of interatrial septum.

## Background

In the late 1950s, Ross et al. introduced transseptal catheterization for evaluating valvular heart disease [1]. Recent advances in this percutaneous procedure have enabled treatment of arrhythmia, valvular and congenital disorders through such interventions to avoid open heart surgery. Nevertheless, a transseptal approach is not without complications, namely persistent iatrogenic atrial septal defects, cardiac tamponade, and rupture [2, 3]. Reported herein is the discovery of a left atrial thrombus found years after transseptal catheterization and clinically misinterpreted as a solid mass of right atrium. The thrombus had formed within a septal aneurysm.

## Case presentation

A 72-year-old female was admitted for left-sided weakness. Magnetic resonance imaging (MRI) of the brain disclosed multifocal acute infarcts of right insular and peri-insular regions, right temporal cortex and ipsilateral postcentral gyrus, and right middle cerebral arterial supply. Vital signs were stable, and electrocardiogram showed atrial fibrillation without rapid ventricular response. The patient unmonitored at the time had undergone percutaneous mitral valvotomy for rheumatic mitral stenosis 7 years earlier at another institute. But, she has not been to the hospital since then and has not taken any medication, including anticoagulants.

**Fig. 1:**
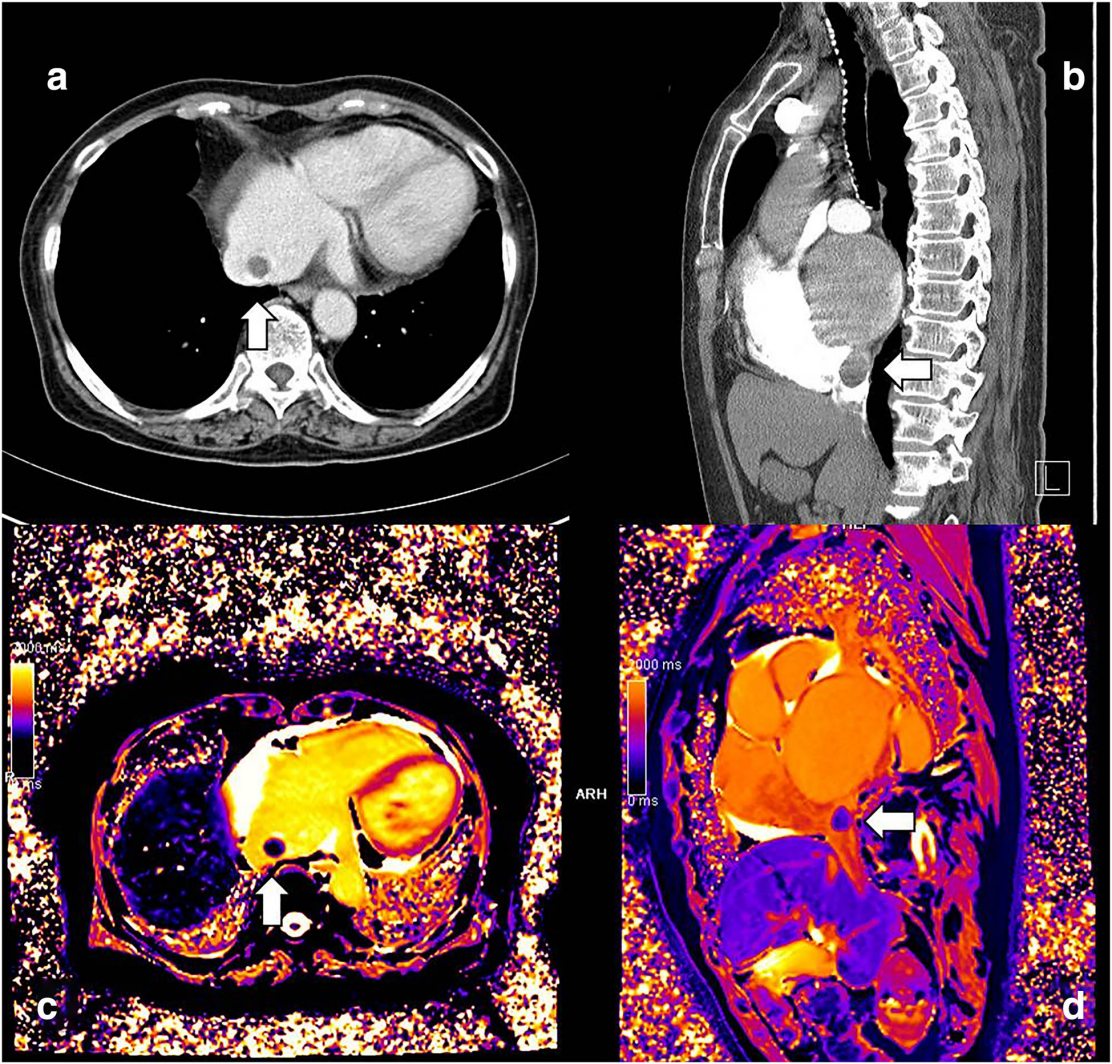
Preoperative computed tomography (**a, b**) and magnetic resonance (**c, d**) studies disclosed a smooth, rounded mass within right atrium (arrows)

Transthoracic echocardiography performed on admission showed normal left ventricular function, with negligible mitral regurgitation, mild mitral stenosis. It revealed a fixed, broad-based 1.5-cm mass with a smooth, rounded surface was identified at the junction of right atrium (RA) and inferior vena cava (IVC). This lesion was not mentioned in the previous examination. A low-density, well-defined ovoid mass was also visible by computed tomography (CT) scan. The radiographic interpretation consequently was solid mass of right atrium, arising from interatrial septum. High signal intensity on both T1- and T2-weighted images, with little or no enhancement on cardiac MRI, suggested a benign cardiac tumor (Fig. 1). Thrombus in the left atrial appendage was not observed in all tests including echocardiography, CT and MRI.

**Fig. 2:**
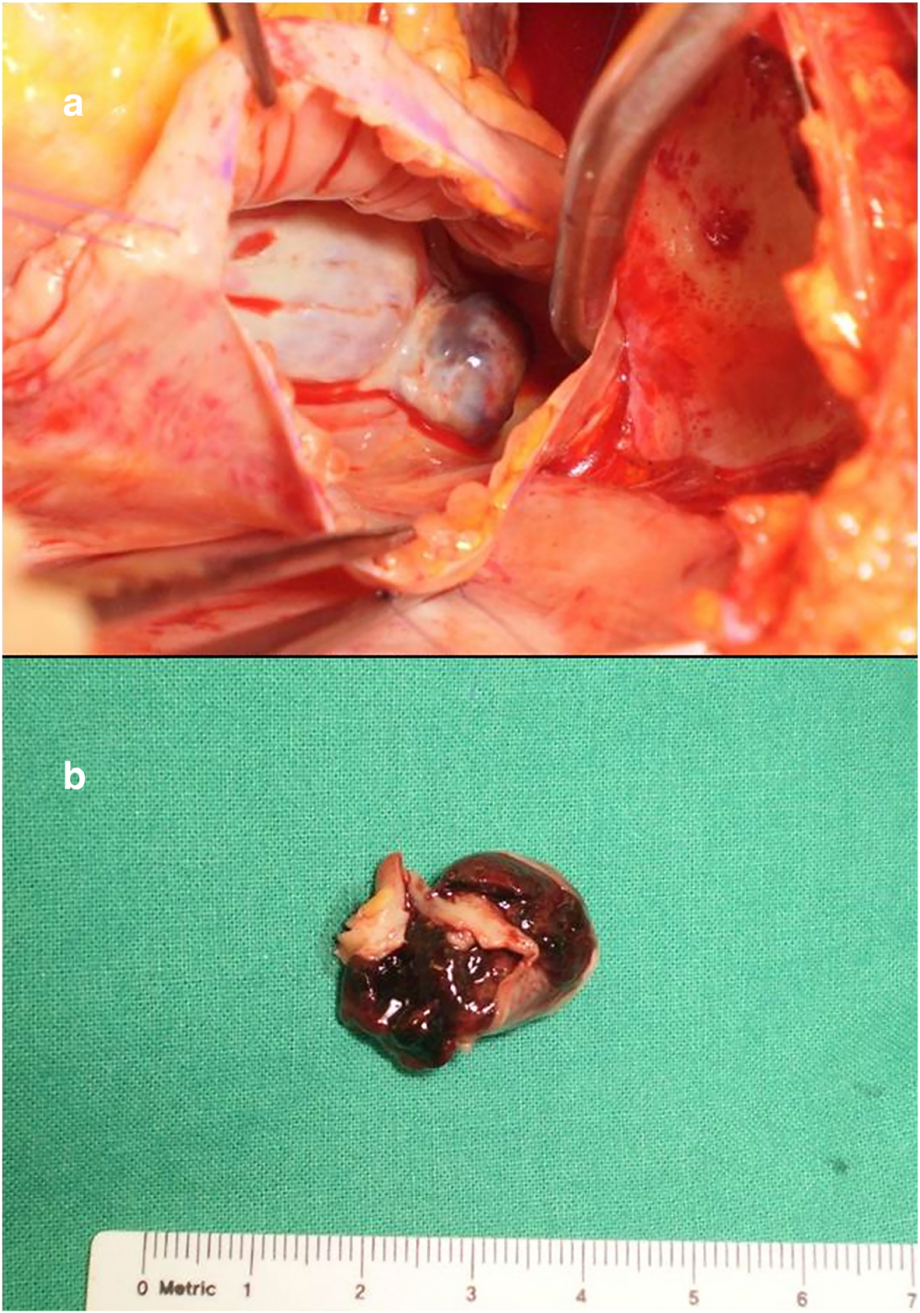
Intraoperatively, the soft and smoothly contoured mass protruding into right atrium (**a**) contained fresh thrombus (**b**)

We decided to remove the right atrial mass, and performed median sternotomy. Under total circulatory arrest, RA was opened, focusing on the location pinpointed in images. The mass was soft, round, and grossly cystic. Once excised along with adjacent atrial septum, fresh thrombus was found inside what proved to be a septal aneurysm of left atrium (LA). In other words, the base of the mass bore a connection to LA and was filled with hemorrhagic clot (Fig. 2).

Interatrial septal repair took place directly thereafter (cardiopulmonary bypass, 207 min; aortic cross-clamping, 28 min; total circulatory arrest, 15 min; rectal temperature, 20 °C). No additional procedures were warranted. The patient was discharged 12 days postoperatively, without complications, and the 2 years since have been uneventful. Final pathology review confirmed a cystic mural thrombus (2 × 1.5 × 1.3 cm), clinically simulating a mass of right atrium.

## Discussion

As percutaneous procedures continue to evolve, transseptal catheterization has become popular for treating arrhythmias or mitral valvular disease. However, certain procedural complications are inherent [2, 3]. The catheter placed inside RA generally enters LA via patent foramen ovale or transseptal puncture. Such iatrogenic septal defects may prove detrimental in some patients.

Atrial septal aneurysm (ASA) is an infrequent finding in adults, linked to systemic embolism on occasion [4]. Furthermore, Aksnes et al. have reported on surgical excision of an ASA after a cerebral embolic episode [5]. The ASA in our patient had gradually filled with thrombus, protruding into RA. Both location and size made it difficult to accurately characterize. Without operative treatment, the exposed fresh thrombus of left atrium virtually ensured recurrent embolic insults. Even gentle intraoperative manipulation may have precipitated immediate postoperative stroke.

## Conclusions

Prior transseptal intervention should be considered in patients presenting with mass lesions of interatrial septum. Required surgical excision calls for extreme care to prevent perioperative systemic embolization.
